# Supplementary material for: Depressive Symptoms of Public Health Medical Residents during the COVID-19 Pandemic, a Nation-Wide Survey: The PHRASI Study
Source: Int J Environ Res Public Health. 2023 Apr 24;20(9):5620. doi: 10.3390/ijerph20095620 (PMC10178174; doi:10.3390/ijerph20095620)
Supplement: Supplementary file 1 [file ijerph-20-05620-s001.zip › ijerph-2164308-supplementary.pdf]

**Figure S1.** Multicollinearity analysis

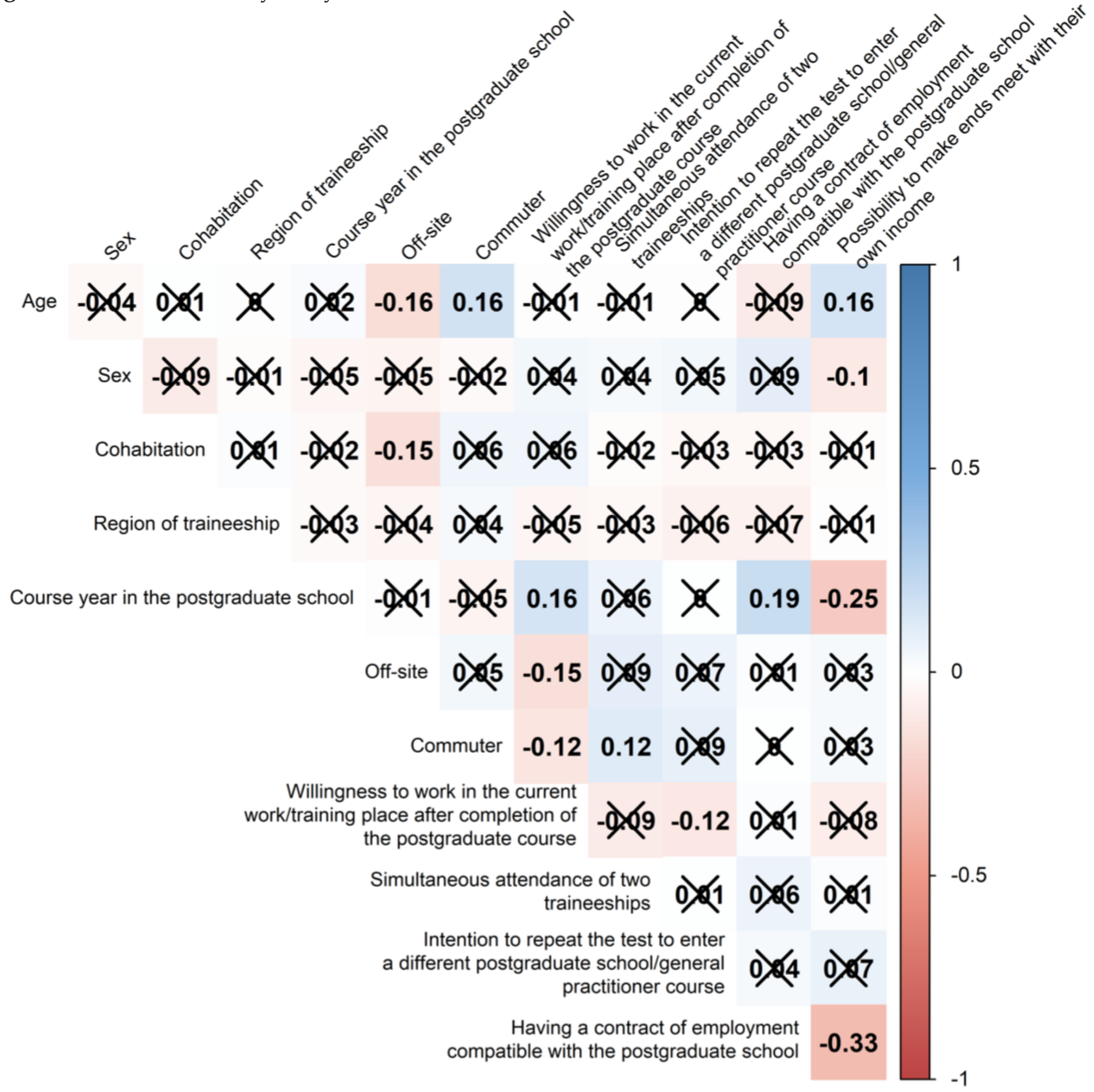


**Table S1.** Relations between socio-demographic characteristics and depressive symptoms (PHQ-9≥5).

| Characteristic | No depressive symptoms (PHQ-9<5) N = 148[1] | Depressive symptoms (PHQ-9≥5) N = 231[1] | p-value[2] |
|---|---|---|---|
| **Age [median, (IQR)]** | 30 (28.00, 34) | 31 (29.00, 33) | 0.4 |
| **Sex** | | | |
| Female | 82 (55.41%) | 137 (59.31%) | 0.5 |
| Male | 66 (44.59%) | 94 (40.69%) | |
| **Region of residence** | | | |
| Center | 41 (27.70%) | 55 (23.81%) | 0.5 |
| North | 56 (37.84%) | 101 (43.72%) | |
| South and islands | 51 (34.46%) | 75 (32.47%) | |
| **Cohabitation** | | | |
| Alone | 37 (25.00%) | 61 (26.41%) | 0.8 |

| | | | |
|---|---|---|---|
| With Others | 111 (75.00%) | 170 (73.59%) | |
| **Number of children** | | | |
| 0 | 129 (87.16%) | 198 (85.71%) | |
| 1 | 11 (7.43%) | 21 (9.09%) | 0.9 |
| >1 | 8 (5.41%) | 12 (5.19%) | |
| **Region of traineeship** | | | |
| Center | 44 (29.73%) | 69 (29.87%) | |
| North | 67 (45.27%) | 111 (48.05%) | 0.8 |
| South | 37 (25.00%) | 51 (22.08%) | |
| **Course year in the postgraduate school** | | | |
| 1st biennium | 120 (81.08%) | 172 (74.46%) | 0.13 |
| 2nd biennium | 28 (18.92%) | 59 (25.54%) | |
| **Off-site** | | | |
| No | 76 (51.35%) | 135 (58.44%) | 0.2 |
| Yes | 72 (48.65%) | 96 (41.56%) | |
| **Commuter** | | | |
| No | 108 (72.97%) | 150 (64.94%) | 0.10 |
| Yes | 40 (27.03%) | 81 (35.06%) | |
| **Willingness to work in the current work/training place after completion of the postgraduate course** | | | |
| No | 38 (25.68%) | 87 (37.66%) | 0.015 |
| Yes | 110 (74.32%) | 144 (62.34%) | |
| **Simultaneous attendance of two traineeships** | | | |
| No | 132 (89.19%) | 189 (81.82%) | 0.052 |
| Yes | 16 (10.81%) | 42 (18.18%) | |
| **Intention to repeat the test to enter a different postgraduate school/general practitioner course** | | | |
| No | 139 (93.92%) | 176 (76.19%) | |
| Maybe | 7 (4.73%) | 39 (16.88%) | <0.001 |
| Yes | 2 (1.35%) | 16 (6.93%) | |
| **Having a contract of employment compatible with the postgraduate school** | | | |
| No | 101 (68.24%) | 141 (61.04%) | 0.2 |

| | | | |
|---|---|---|---|
| Yes | 47 (31.76%) | 90 (38.96%) | |
| **Capacity to make ends meet with the own income** | | | |
| Easily | 72 (48.65%) | 90 (38.96%) | 0.063 |
| Hardly | 76 (51.35%) | 141 (61.04%) | |

[1] Median (IQR); n (%)

[2] Wilcoxon rank sum test; Pearson's Chi-squared test

**Table S2.** Multivariate logistic regression adjusted for age, sex and for each single variable (PQH-9≥5).

| Variable | aOR related to depressive symptoms (PHQ≥5) | 95% CI | p-value |
|---|---|---|---|
| **Region of traineeship (ref. Center)** | | | |
| North | 1.056 | 0.649 - 1.719 | 0.826 |
| South and islands | 0.877 | 0.497 - 1.548 | 0.651 |
| **Cohabitation (ref. Alone)** | | | |
| With Others | 0.912 | 0.567 - 1.468 | 0.706 |
| **Off-site (ref. No)** | | | |
| Yes | 0.749 | 0.491 - 1.142 | 0.180 |
| **Commuter (ref. No)** | | | |
| Yes | 1.443 | 0.912 - 2.282 | 0.117 |
| **Willingness to work in the current work/training place after completion of the postgraduate course (ref. No)** | | | |
| Yes | 0.575 | 0.365 - 0.907 | 0.017 |
| **Simultaneous attendance of two traineeships (ref. No)** | | | |
| Yes | 1.853 | 0.999 - 3.440 | 0.050 |
| **Intention to repeat the test to enter a different postgraduate school/general practitioner course (ref. No)** | | | |

| | | | |
|---|---|---|---|
| Maybe | 4.453 | 1.930 - 10.275 | <0.001 |
| Yes | 6.475 | 1.461 - 28.689 | 0.014 |
| **Having a contract of employment compatible with the postgraduate school (ref. No)** | | | |
| Yes | 1.414 | 0.910 - 2.195 | 0.123 |
| **Capacity to make ends meet with their own income (ref. Easily)** | | | |
| Hardly | 1.458 | 0.954 - 2.228 | 0.082 |

**Table S3.** Relations between socio-demographic characteristics and depressive symptoms (PHQ-9≥5), stratified by sex.

| | **Female** | | | **Male** | | |
|---|---|---|---|---|---|---|
| **Characteristic** | **No depressive symptoms (PHQ-9<5) N = 82**[1] | **Depressive symptoms (PHQ-9≥5) N = 137**[1] | **p-value**[2] | **No depressive symptoms (PHQ-9<5) N = 66**[1] | **Depressive symptoms (PHQ-9≥5) N = 94**[1] | **p-value**[2] |
| **Age** [median, (IQR)] | 30 (28.00, 34) | 31 (29.00, 34) | 0.2 | 30 (29.00, 34) | 30 (29.00, 33) | 0.7 |
| **Region of residence** | | | | | | |
| Center | 20 (24.39%) | 33 (24.09%) | | 21 (31.82%) | 22 (23.40%) | |
| North | 36 (43.90%) | 62 (45.26%) | >0.9 | 20 (30.30%) | 39 (41.49%) | 0.3 |
| South and islands | 26 (31.71%) | 42 (30.66%) | | 25 (37.88%) | 33 (35.11%) | |
| **Cohabitation** | | | | | | |
| Alone | 18 (21.95%) | 31 (22.63%) | >0.9 | 19 (28.79%) | 30 (31.91%) | 0.7 |
| With Others | 64 (78.05%) | 106 (77.37%) | | 47 (71.21%) | 64 (68.09%) | |
| **Number of children** | | | | | | |
| 0 | 69 (84.15%) | 112 (81.75%) | | 60 (90.91%) | 86 (91.49%) | |
| 1 | 7 (8.54%) | 16 (11.68%) | 0.8 | 4 (6.06%) | 5 (5.32%) | >0.9 |
| >1 | 6 (7.32%) | 9 (6.57%) | | 2 (3.03%) | 3 (3.19%) | |

| | | | | | | |
|---|---|---|---|---|---|---|
| **Region of traineeship** | | | | | | |
| Center | 23 (28.05%) | 39 (28.47%) | | 21 (31.82%) | 30 (31.91%) | |
| North | 39 (47.56%) | 69 (50.36%) | 0.9 | 28 (42.42%) | 42 (44.68%) | >0.9 |
| South and islands | 20 (24.39%) | 29 (21.17%) | | 17 (25.76%) | 22 (23.40%) | |
| **Course year in the Italian specialization school** | | | | | | |
| 1st biennium | 63 (76.83%) | 102 (74.45%) | 0.7 | 57 (86.36%) | 70 (74.47%) | 0.067 |
| 2nd biennium | 19 (23.17%) | 35 (25.55%) | | 9 (13.64%) | 24 (25.53%) | |
| **Off-site** | | | | | | |
| No | 37 (45.12%) | 80 (58.39%) | 0.057 | 39 (59.09%) | 55 (58.51%) | >0.9 |
| Yes | 45 (54.88%) | 57 (41.61%) | | 27 (40.91%) | 39 (41.49%) | |
| **Commuter** | | | | | | |
| No | 56 (68.29%) | 91 (66.42%) | 0.8 | 52 (78.79%) | 59 (62.77%) | 0.030 |
| Yes | 26 (31.71%) | 46 (33.58%) | | 14 (21.21%) | 35 (37.23%) | |
| **Willingness to work in the current work/training place after completion of the postgraduate course** | | | | | | |
| No | 22 (26.83%) | 54 (39.42%) | 0.058 | 16 (24.24%) | 33 (35.11%) | 0.14 |
| Yes | 60 (73.17%) | 83 (60.58%) | | 50 (75.76%) | 61 (64.89%) | |
| **Simultaneous attendance of two traineeships** | | | | | | |
| No | 73 (89.02%) | 115 (83.94%) | 0.3 | 59 (89.39%) | 74 (78.72%) | 0.076 |
| Yes | 9 (10.98%) | 22 (16.06%) | | 7 (10.61%) | 20 (21.28%) | |
| **Intention to repeat specialization schools/general practitioner test** | | | | | | |

| | | | | | | |
|---|---|---|---|---|---|---|
| No | 79 (96.34%) | 106 (77.37%) | | 60 (90.91%) | 70 (74.47%) | |
| Maybe | 2 (2.44%) | 23 (16.79%) | <0.001 | 5 (7.58%) | 16 (17.02%) | 0.029 |
| Yes | 1 (1.22%) | 8 (5.84%) | | 1 (1.52%) | 8 (8.51%) | |
| **Having a contract of employment compatible with the postgraduate school** | | | | | | |
| No | 58 (70.73%) | 90 (65.69%) | 0.4 | 43 (65.15%) | 51 (54.26%) | 0.2 |
| Yes | 24 (29.27%) | 47 (34.31%) | | 23 (34.85%) | 43 (45.74%) | |
| **Capacity to make ends meet with their own income** | | | | | | |
| Easily | 40 (48.78%) | 44 (32.12%) | 0.014 | 32 (48.48%) | 46 (48.94%) | >0.9 |
| Hardly | 42 (51.22%) | 93 (67.88%) | | 34 (51.52%) | 48 (51.06%) | |

[1] Median (IQR); n (%)

[2] Wilcoxon rank sum test; Pearson's Chi-squared test; Fisher's Exact Test for Count Data with simulated p-value (based on 10000 replicates)

**Table S4.** Negative binomial regression adjusted for sex and age.

| Variable | aRR | 95% CI | p-value |
|---|---|---|---|
| **Cohabitation (ref. Alone)** | | | |
| With Others | 0.854 | 0.72 - 1.012 | 0.069 |
| **Region of traineeship (ref. Center)** | | | |
| North | 1.030 | 0.863 - 1.229 | 0.745 |
| South | 1.011 | 0.821 - 1.245 | 0.921 |
| **Off-site (ref. No)** | | | |
| Yes | 1.022 | 0.877 - 1.192 | 0.779 |
| **Commuter (ref. No)** | | | |
| Yes | 1.067 | 0.907 - 1.256 | 0.435 |
| **Willingness to work in the current work/training place after completion of the postgraduate course (ref. No)** | | | |

| | | | |
|---|---|---|---|
| Yes | 0.754 | 0.646 - 0.882 | 0.000 |
| **Simultaneous attendance of two traineeships (ref. No)** | | | |
| Yes | 1.288 | 1.051 - 1.578 | 0.015 |
| **Intention to repeat the test to enter a different postgraduate school/general practitioner course (ref. No)** | | | |
| Maybe | 1.686 | 1.364 - 2.085 | 0.000 |
| Yes | 1.677 | 1.212 - 2.321 | 0.002 |
| **Having a contract of employment compatible with the postgraduate school (ref. No)** | | | |
| Yes | 1.051 | 0.898 - 1.231 | 0.535 |
| **Possibility to make ends meet with their own income (ref. Easily)** | | | |
| Hardly | 1.132 | 0.97 - 1.321 | 0.116 |
